# Effect of microencapsulation of egg yolk immunoglobulin Y by sodium alginate/chitosan/sodium alginate on the growth performance, serum parameters, and intestinal health of broiler chickens

**DOI:** 10.5713/ab.22.0414

**Published:** 2023-02-27

**Authors:** Yuanming Jin, Haojie Lv, Mingzhu Wang, Chong-Su Cho, Jongsuh Shin, Lianhua Cui, Changguo Yan

**Affiliations:** 1 Department of Animal Science, Yanbian University, Yanji, Jilin 133002, China; 2 Engineering Research Center of North-East Cold Region Beef Cattle Science and Technology Innovation, Ministry of Education, Yanbian University, Yanji, Jilin 133002, China; 3 Department of Agricultural Biotechnology and Research Institute of Agriculture and Life Sciences, Seoul National University, Seoul 08826, Korea; 4 Department of Animal Life Sciences, Kangwon National University, Chuncheon 24341, Korea; 5 Yanbian Hongchao Smart Animal Husbandry Co., Ltd, Yanji, Jilin 133002, China

**Keywords:** Broiler, Immunoglobulin Y (IgY), Immune Performance, Intestinal Flora, Intestinal Morphology, Microencapsulation, Production Performance

## Abstract

**Objective:**

Egg yolk immunoglobulin (IgY) is an antibiotic alternative to prevent and fight intestinal pathogenic infections. This study aimed to investigate the effects of sodium alginate/chitosan/sodium alginate IgY microcapsules on the growth performance, serum parameters, and intestinal health of broiler chickens.

**Methods:**

One-day-old broilers (Ross 308) were divided into five treatments, each with 10 replicates of five chickens. The dietary treatments were maintained for 28 days and consisted of a basal diet (NC), basal diet + 500 mg chlortetracycline/kg diet (CH), basal diet + 50 mg non-microencapsulated IgY/kg diet (NM), basal diet + 600 mg low levels microencapsulated IgY/kg diet (LM), and basal diet + 700 mg high levels microencapsulated IgY/kg diet (HM).

**Results:**

Throughout the 28-day trial period, the NM, LM, HM, and CH groups increased average daily gain compared with the NC group (p<0.05), and the HM group reduced feed conversion ratio compared with the CH group (p<0.05). The LM and HM groups increased relative organ weights of thymus and spleen compared with the CH and NM groups (p< 0.05). The HM group improved the duodenal, jejunal and ileum villi height (VH) and villus height to crypt depth ratio (VH:CD) compared with the CH and NM groups (p<0.05). Compared with the CH group, the HM group increased serum immunoglobulin (IgA), immunoglobulin G (IgG), superoxide dismutase, total antioxidant capacity, and glutathione peroxidase levels (p<0.05), and decreased serum malondialdehyde levels (p<0.05). Compared with the NC group, the NM, LM, HM, and CH groups reduced colonic *Escherichia coli* and Salmonella levels (p<0.05). and the HM group promoted the levels of lactic acid bacteria and bifidobacteria compared with the CH group (p<0.05).

**Conclusion:**

Microencapsulation could be considered as a way to improve the efficiency of IgY. The 700 mg high levels microencapsulated IgY/kg diet could potentially be used as an alternative to antibiotics to improve the immune performance and intestinal health, leading to better performance of broiler chickens.

## INTRODUCTION

Intestinal health is critical to the productive performance of livestock and poultry. The immaturity of the immune system of chicks leads to the infestation of pathogenic bacteria such as Salmonella, which affects the performance of broilers [1–3]. Antibiotics are often used for the prevention and treatment of pathogens, but their long-term use can cause negative effects, such as bacterial resistance, imbalances of animal intestinal flora, and drug residues, posing a serious threat to animal and human health [4]. Therefore, in order to prevent the negative effects of antibiotic abuse, to meet the growing consumer demand for high quality livestock products, there is an urgent need to develop effective, safe, economical, and eco-friendly alternatives to antibiotics.

Egg yolk immunoglobulin, also known as immunoglobulin Y (IgY), is an antigen-specific antibody produced by B lymphocytes that accumulate in egg yolk. Based on this physiological mechanism, IgY can be artificially obtained from the yolk [5]. Recently, IgY has received considerable attention as a means of disease control because of its cost-effectiveness, convenience, and high yield [6]. Overall, it is a promising alternative to antibiotics. Numerous studies have shown that IgY can bind to specific sites on enterotoxin-producing *Escherichia coli* (*E. coli*), Salmonella, and other intestinal pathogens to prevent them from adhering to the small intestinal mucosa or to inhibit their growth and reproduction, thus preventing the occurrence of intestinal infections [7,8]; however, IgY is susceptible to degradation by pepsin and loss of antibody activity in a high-intensity gastric acid environment [9,10], but antibodies generally need to survive in the gastrointestinal environment and reach their target area with their biological properties intact [11]. Therefore, effective methods must be adopted to protect IgY from pepsin degradation and gastric acid environment to maximize the preservation of the biological activity of orally administered IgY.

Microencapsulation provides a protective barrier against adverse conditions and can enhance stability in harsh environments [12]. The varying pH and enzymatic environments in the stomach and intestine require the wall material of microcapsules (MCs) to have pH-sensitivity and swelling properties to control the release of the core material [13,14]. Studies have shown that sodium alginate (SA) and chitosan (CS) are biocompatible, biodegradable, non-toxic, and uniquely pH-responsive, and have been increasingly used for encapsulation and stabilization in enteral drug delivery systems [15–17]. While combining SA and CS has been effective, in some in vitro studies, combined SA/CS MCs are poorly tolerated by gastric acid because the residual carboxyl group tends to break the ionic bond linkage when exposed to water. Some scholars have found that SA/CS/SA encapsulation can be used to improve its tolerance; for example, Cui et al [18] prepared SA/CS/SA MCs to successfully encapsulate probiotics using pH-sensitive materials, CS, and SA as wall materials, which enhanced the tolerance of the probiotic gastrointestinal tract, and Jiang et al [19] encapsulated a probiotic expression vaccine in SA/CS/SA MCs, to enhance its survival in simulated gastrointestinal fluids.

Given the adverse effects of antibiotic use and the poor gastrointestinal tolerance of IgY, new antimicrobial strategies are urgently needed. This study applied SA/CS/SA MCs to load IgY for investigating the effects of microencapsulation of IgY by SA/CS/SA on the growth performance, serum parameters, and intestinal health of broiler chickens.

## MATERIALS AND METHODS

### Animal care

The animal experiments involved in this experiment strictly followed the requirements in the Guidelines for the Ethics and Use of Agricultural Animals in Research and Teaching [20] and were reviewed by the Experimental Animal Ethics Committee of Yanbian University (ethical review acceptance number: YD20220718003).

### Preparation of immunoglobulin Y

IgY was prepared as described in previous research [21,22]. High-quality eggs with yolk contents of 100 mg or more were selected, and the yolk was precipitated with ammonium sulfate. IgY was slowly precipitated from the yolk by dilution with sterile water and purified using the low-temperature ethanol method. Additionally, the temperature was lowered to −20°C to prevent protein denaturation and remove excess ethanol. After salt precipitation, IgY of >90% purity was obtained and converted to a powder by heating before being stored at −20°C.

### Microencapsulation of immunoglobulin Y by SA/CS/SA

**Figure 1:**
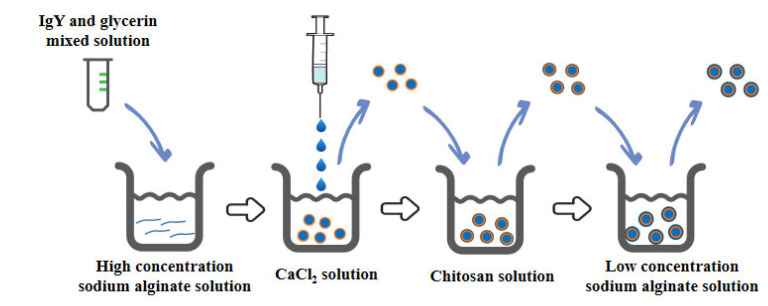
Schematic diagram of the preparation of sodium alginate/chitosan/sodium alginate immunoglobulin Y microcapsules.

Microcapsules were prepared as described by Cui et al [18], with a mixture of IgY and glycerol injected into a highly concentrated SA solution and stirred well (Figure 1). The mixture of SA with IgY and glycerol was dropped into a 0.1 M CaCl2 solution using a syringe under nitrogen pressure. The calcium ions caused the SA droplets to gel and immediately form SA MCs at a concentration of 1.5% (w/v) of SA. The above obtained MCs were stirred uniformly in 0.1 M CaCl2 solution for 30 min, and then washed with 0.85% sterile saline. Then it was further encapsulated with 0.8% CS solution for 30 min and then washed with sterile saline to remove the residual solution. Finally, it was encapsulated with 0.1% low concentration SA solution for 10 min and washed. The prepared samples were stored frozen at −70°C and the MCs were obtained after freeze-drying.

### Experimental design, diets, and husbandry

All animal experiments were conducted at the teaching ranch of the College of Agriculture, Yanbian University.

One-day-old broilers (Ross 308) free of any known pathogen challenges were divided into five treatments, each with 10 replicates of five chickens. The 28-day dietary treatments included basal diet (NC), basal diet + 500 mg chlortetracycline/kg diet (CH), basal diet + 50 mg non-microencapsulated IgY/kg diet (NM), basal diet + 600 mg low levels microencapsulated IgY/kg diet (net addition of IgY was 50 mg/kg) (LM), and basal diet + 700 mg high levels microencapsulated IgY/kg diet (net addition of IgY was 60 mg/kg) (HM). The basic diet in Table 1 was prepared with reference to the nutritional requirements of the Chinese Chicken Feed Standard (NY/T 33-2004).

### Immunization

Newcastle disease IV vaccine (Beijing Centrebio Biological Co., Ltd, Beijing, China) was used for nasal drip immunization at 7 days old. Newcastle disease, infectious bronchitis, and avian influenza (ND-IB-AI) triple-inactivated vaccines (Beijing Centrebio Biological Co., Ltd, China) were used for subcutaneous vaccination.

### Growth performance parameters and sampling

Growth performance parameters (e.g., feed intake [FI] and body weight [BW]) of broilers were recorded daily, and then average daily gain (ADG), average daily feed intake (AFI) and feed conversion ratio (FCR) were calculated.

On day 28, 10 broilers (1 chicks from each replicate) were randomly selected. Blood samples were collected in 10 mL blood collection tubes for serum biochemistry, immunoglobulin and antioxidant index. Subsequently, colonic contents and feces were collected in a sterile environment and stored at −20°C. Duodenal segments, jejunal segments and ileal segments of each sample were collected and stored in formalin fixative (10 vol-%) for intestinal histomorphometric studies.

### Relative organ weights and intestinal lengths

The lengths of duodenum, jejunum, and ileum were measured and calculated, while organ weights such as thymus, spleen, and bursa phalloides were weighed to calculate relative organ weights.

The calculation formula is as follows:


Relative organ weight (g/kg)=organ weight (g)/live BW (kg)

Relative intestinal length (cm/kg)=intestinal length (cm)/live BW (kg)

### Blood biochemistry

The collected blood samples were centrifuged at 5,000×g for 10 minutes at 4°C, and the serum samples were sent to Beijing Sino-UK Institute of Biological Technology for blood biochemical analysis, where serum samples were analyzed biochemically using the Cobas 6000 analyzer series (Roche Diagnostics, Indianapolis, IN, USA).

### Serum antioxidants and Immunoglobulins

Malondialdehyde (MDA) content was determined by thiobarbituric acid method; glutathione peroxidase (GSH-Px) content was determined by microenzyme method; superoxide dismutase (SOD) was determined by xanthine oxidase method (hydroxylamine method), and total antioxidant capacity (T-AOC) was determined by total antioxidant capacity test kit (ABTS method). The kits used for the determination of these indicators were purchased from Nanjing Jiancheng Institute of Biological Engineering and used with reference to the instructions. Serum immunoglobulin A (IgA), immunoglobulin M (IgM), and immunoglobulin G (IgG) levels were determined by the immunoturbidimetric method from the Beijing Sino-UK Institute of Biological Technology, Beijing, China.

### Intestinal morphology

Intestinal tissues stored in fixative were embedded in paraffin, and sections with a thickness of 0.5 μm were cut and subsequently stained with hematoxylin and eosin. The prepared paraffin section samples were brought to the microscope, and the villi height (VH) (from the base of the intestinal mucosa to the tip of the villi, excluding the intestinal crypt) and crypt depth (CD) (from the base to the transition zone between the crypt and the villi) were determined for each sample using a 100× magnification and an image processing analysis system (Olympus BX5, Tokyo, Japan), and the ratio of villi height to crypt depth (VH:CD) was calculated.

### Bacterial population

The contents were thawed naturally in a sterile environment, and approximately 0.1 g of the sample was weighed and dissolved in 0.9 mL of sterilized 0.01 M phosphate buffered solution, mixed, and set aside. Salmonella was used as a hektoen enteric (HE) agar selective medium, E. coli was used as a MacConkey agar selective medium, Lactobacillus was used as a Man-Rogosa-Sharpe (MRS) agar selective medium, and Bifidobacterium was used as a BBL agar selective medium. E. coli, salmonella, lactobacillus, and bifidobacterium cultures were incubated in a constant-temperature incubator at 37°C until complete colonies appeared. The colonies of the bacterial population were expressed as log10 colony forming units per gram of fresh content.

### Statistical analysis

Statistical analyses were performed using SPSS17.0 software. The data were analyzed using one-way analysis of variance for dietary treatment as the primary variation source. Significant differences among the means of the treatments were compared using Tukey’s test, and differences were considered statistically significant at p≤0.05.

## RESULTS

### Growth performance

The effects of the IgY-loaded SA/CS/SA MCs on broiler growth performance are shown in Table 2. At the end of the experiment, the BW increased significantly in all groups compared to that in the NC group. From days 1 to 14 of the experiment, the differences in ADG, AFI, and FCR between the groups were not statistically significant. From days 14 to 28 of the experiment, ADG and FCR were significantly improved in the CH group and different IgY treatment groups relative to the NC group (p<0.05); throughout the experimental cycle, ADG was significantly improved in all treatment groups compared with the NC group (p<0.05), and FCR was significantly lower in the HM group compared with the CH group (p<0.05).

### Relative organ weights and intestinal lengths

The effects of the IgY-loaded SA/CS/SA MCs on the relative organ weights and intestinal lengths in broilers are shown in Table 3. The relative organ weights of thymus and spleen were significantly increased in the LM and HM groups compared with the NC group (p<0.05). with similar effects in the NM and CH groups (p>0.05); however, no significant differences were seen in the relative lengths of duodenum, jejunum and ileum, as well as the relative weight of the bursa of fabricius, between broilers in the different treatments (p>0.05).

### Intestinal morphology

The effects of the IgY-loaded SA/CS/SA MCs on the morphology of broiler intestinal segments are shown in Table 4. In the duodenal segment, the CH, LM, and HM groups had higher VH in the broilers than the NC group (p<0.05), with the LM and HM groups, also increased VH:CD in the broilers (p<0.05). In the jejunal and ileal segments, LM and HM increased VH and VH:CD in the broilers compared with the NC group (p<0.05). In all of the intestinal segments, VH:CD was significant in the HM group than in the CH and NM groups (p<0.05). Notably, there was no significant difference in CD between treatments in each intestinal segment (p>0.05).

### Serum biochemical

The effects of the IgY-loaded SA/CS/SA MCs on the serum biochemical indices of broiler chickens are shown in Table 5. There were no significant differences in serum total protein (TP), total cholesterol (TC), triglyceride (TG), glucose (GLU), and alkaline phosphatase (ALP) levels between treatment groups (p>0.05). Compared to the NC group, the CH, LM, and HM groups had significantly higher albumin (ALB) levels (p<0.05), with the LM and HM groups having significantly higher ALB levels than the NM group (p<0.05). The effect in the NM group was similar to that in the NC and CH groups (p>0.05).

### Serum immunoglobulins

The effects of the IgY-loaded SA/CS/SA MCs on serum immunoprotein levels in the broiler chickens are shown in Table 6. Compared with the NC group, each treatment group showed improved serum immunoprotein levels to different degrees (p<0.05). The HM group showed significantly improved serum IgA and IgG levels compared to the CH group (p<0.05). Compared with NC group, serum IgM levels in NM, LM and HM groups was significantly increased (p<0.05), while the CH and NC groups had similar effects (p>0.05).

### Serum antioxidant

The effects of the IgY-loaded SA/CS/SA MCs on serum antioxidants in the broiler chickens are shown in Table 7. Compared with NC group, SOD, GSH-Px, and MDA levels in all treatment groups were significantly increased (p<0.05). The best results were obtained in the HM group compared with the CH group (p<0.05). Notably, T-AOC levels were significantly higher in the HM group than those in the other treatment groups (p<0.05).

### Bacterial population

The effects of the IgY-loaded SA/CS/SA MCs on the intestinal bacterial flora of the broiler chickens are shown in Table 8. Colonic content levels of *E. coli* and Salmonella were significantly lower in each treatment group than in the NC group (p<0.05). The levels of lactic acid bacteria were significantly increased in the LM and HM groups (p<0.05), and only Bifidobacterium was significantly increased in the HM group (p<0.05).

## DISCUSSION

Oral chicken yolk antibodies (IgY) represent an emerging and promising immune strategy for infection control in the broiler industry [23]. The addition of IgYs to diets has been shown by Mahdavi et al [24] to increase the average weight gain and feed conversion ratio in broilers, whereas the growth-promoting properties of antibiotics have been well established globally [25–27]. Our experimental results showed that the addition of different IgY and antibiotic treatments to the feed significantly increased ADG and significantly reduced FCR, suggesting that more chicken meat can be produced when the same amount of feed is consumed, effectively increasing energy conversion and protein deposition [28]. We found that adding high levels of microencapsulated IgY to livestock diets resulted in a better reduction in FCR than that caused by antibiotic-inclusive diets. Generally, a lower FCR is important in promoting sustainable animal husbandry and reducing environmental pollution [29]. Our results also showed that high levels of microencapsulated IgY were significantly effective compared to low levels of microencapsulated IgY. This is consistent with Attia et al [30] who reported that the composition and dose of active ingredients in the feed, animal factors and experimental environment affect their growth performance. We conclude that adding high levels of microencapsulated IgY to livestock diets as antibiotic replacements can reduce the environmental impact of antibiotics and their metabolites and improve the growth performance of broilers.

Thymus, spleen and bursa of Fabricius are important immune organs of broilers. The development of these immune organs is particularly important for the immune level of the body and the ability to resist foreign microbial infection and invasion. An increase in their absolute and relative weights indicates enhanced cellular and humoral immune functions of the organism [31,32]. Our results showed that microencapsulated IgY significantly increased the relative weights of the spleen and thymus in the broilers, suggesting that microencapsulated IgY could better promote the growth and development of immune organs in the broilers. It can be used as an antigenic substance to promote immune organ development, thereby strengthening the overall immune function of broilers and improving their ability to resist various pathogenic microbial infections and stresses.

Small intestinal VH and CD are important indicators of changes in intestinal physiology. An increase in villus height increases the efficiency of nutrient absorption, and similarly, an increase in VH:CD can increase energy reserves [33,34]. In this study, VH and VH:CD improved in all segments of the small intestine in both the microencapsulated IgY and antibiotic groups. Previous studies have demonstrated that IgY and antibiotic addition to the diets can improve the development of broiler intestinal villi [24,35]. Moreover, the significant effect of microencapsulated IgY over non-microencapsulated IgY in this experiment may be due to the denaturation of non-microencapsulated IgY in the acidic environment of the stomach and its degradation by protein hydrolases present in the stomach and small intestine [14], leading to the increased digestibility of IgY. Consequently, the amount of IgY reaching the intestine is relatively limited. In contrast, the SA/CS/SA protection improved the stability of IgY. Zhang et al [36] reported that compared with non-microencapsulated IgY, IgY MCs maintained 84.37% immunoreactivity after 4 h in a simulated digestive environment [36]. Thus, under the influence of the gastric acid environment, the outer layer of microencapsulated IgY gradually swells and dissolves, while the inner layer remains under acidic conditions, which helps IgY resist the action of endogenous enzymes, thus improving its release properties in the duodenum and jejunum terminals. The positive effect of microencapsulated IgY on VH in this experiment may reflect the increased concentration of IgY reaching the small intestine [37,38]. Thuekeaw et al [39]. reported that adding 500 ppm of microencapsulated basil oil to their diets promoted VH:CD in the studied broilers compared with free basil oil [39]. Therefore, microencapsulated IgY additions to livestock diets can improve the structure of small intestinal villi and promote the absorption and utilization of nutrients in the small intestine of broiler chickens.

Blood biochemistry is important in the detection of animal organisms, and changes in its composition can reflect the metabolic and health status of the animal. The serum biochemical level measured in this test is within the normal physiological range [40,41]. TP, TC, TG, GLU, and ALP levels did not vary significantly among the groups, and ALB levels were significantly affected by dietary treatment. Studies have shown that serum ALB can maintain the colloid osmotic pressure and vascular endothelial integrity of body tissues and plays an important physiological role by binding to various endogenous and exogenous compounds and has good anti-inflammatory and free radical elimination effects in the body [42]. In this study, the microencapsulated IgY and antibiotic groups demonstrated similar effects in increasing ALB levels. The effect of microencapsulated IgY was stronger than that of non-microencapsulated IgY.

IgA, IgG, and IgM are important immunoglobulins in poultry, commonly found in the blood, tissue fluids, and exocrine fluids, which are important in infection by foreign pathogen resistances and can reflect the immune level of the organism [43–45]. Previous studies have shown that the addition of certain concentrations of IgY to diets can improve serum IgA and jejunal IgA levels in broilers infected with *E. coli* [24]. In this study, compared to non-microencapsulated IgY, high-dose microencapsulated IgY significantly increased IgA and IgG levels and was significantly more effective than antibiotics.

Oxidative stress is an imbalance between antioxidants and free radicals that generates various reactive oxygen species (ROS) in the body. T-AOC, SOD, and GSH-Px activities in the blood are important indicators of the antioxidant performance in scavenging excess ROS in the body and maintaining a stable and healthy state [46,47]. Studies have shown that oxidative stress can cause gastrointestinal infections and decrease production performance in poultry [48–50]. In this study, different IgY and antibiotic treatments significantly improved serum SOD and GSH-Px levels and significantly reduced MDA levels. Among these, high doses of microencapsulated IgY significantly increased serum T-AOC levels. Generally, microencapsulation reduces IgY loss in the gastrointestinal environment. Similar studies have shown that CS particles can better retain their antioxidant activity compared with the free form of Butyrospermum epoxide [51]. In this experiment, the effect of low-dose microencapsulated IgY was similar to that of non-microencapsulated IgY, suggesting that arrival in the small intestine requires a certain concentration of IgY to be effective.

Intestinal flora is an integral part of intestinal composition. Bifidobacteria and Lactobacillus in the intestine of poultry can bind to surface-specific receptors on the intestinal mucosa to form a bacterial film structure and biological barrier with a fixed composition, which can defend against pathogenic bacteria [52]. The results of this experiment showed that different treatments in both the IgY and antibiotic groups significantly inhibited *E. coli* and Salmonella colonization in the cecum of broiler chickens, and Hatamzade Isfahani et al [53] demonstrated that microencapsulated IgY significantly reduced Salmonella colonization in the intestines of chickens. Han et al [54] showed that anti-ETEC IgY canreduced Enterotoxigenic *Escherichia coli* (ETEC) colonization in the intestine of mice by feeding anti-ETEC IgY and showed that high and medium doses of anti-ETEC IgY provided better overall protection against ETEC infection. Additionally, microencapsulated IgY significantly promoted Lactobacillus and Bifidobacterium populations in the colon. According to Kim et al [55], feeding diets treated with oligofructose (2.5 g/kg diet) increased intestinal Lactobacillus and limited *E. coli* and Clostridium perfringens. The CS used in this experiment can be absorbed and utilized by the intestinal probiotic flora as a prebiotic; therefore, microencapsulated IgY can better promote the value-added of beneficial bacteria and inhibit the proliferation of harmful bacteria to ensure the health of the intestinal structure.

## CONCLUSION

We conclude that microencapsulated IgY increases the efficiency of IgY passage through the gastrointestinal environment compared to non-microencapsulated IgY, which has a positive effect on improving intestinal health and modulating the immune response. The overall effect of adding 700 mg high levels microencapsulated IgY/kg diet to poultry diets was better than that of other IgY treatment groups and antibiotic groups could potentially be used as an alternative to improve the immune performance and intestinal health, leading to better performance of broiler chickens.

## Figures and Tables

**Table 1:** Composition and nutritional level of basal diet

Items	Composition (%)
Ingredient	
Corn	64.29
Soybean meal	27.55
Corn protein powder	3.1
Salt	0.26
Calcium hydrogen phosphate	1.49
Mineral meal	0.11
Bran	2.2
Vitamin and mineral premixes^1)^	1
Total	100
Calculated chemical composition^2)^	
Metabolic energy (MJ/kg)	2.83
Crude protein	20.95
Lysine	0.92
Methionine	0.32
Calcium	0.59
Total phosphorus	0.73
Non-phytate phosphorus	0.48

1) Provided the following quantities per kg of complete diet: vitamin A 10,999 IU; vitamin D_3_ 3,000 IU; vitamin E 15 IU; vitamin K 319 mg; vitamin B 110 mg; vitamin B_2_ 30 mg; vitamin B_6_ 20 mg; vitamin B_12_ 0.2 mg; nicotinic acid 599 mg; pantothenic acid 181 mg; folic acid 10.1 mg; biotin 0.79 mg; Choline 6.99 mg; Cu 0.2 g; Fe 1.2 g; Mn 1.9 g; Zn 1.8 g; I 10 mg; Se 5.99 mg.

2) Nutrient levels are calculated values.

**Table 2:** The effects of the IgY-loaded SA/CS/SA microcapsules on broiler growth performance ^1)^

Item	Dietary treatment^2)^					SEM	p-value

	NC	CH	NM	LM	HM		
Initial body weight (g)	45.6	44.0	45.7	44.6	44.2	0.488	0.735
Final body weight (g)	1,079.3^b^	1,220.9^a^	1,249.9^a^	1,215.1^a^	1,287.1^a^	15.057	<0.001
Day 1 to 14							
Average daily gain (g/d)	18.98	19.22	20.06	19.42	20.52	0.380	0.708
Average daily feed intake (g/d)	35.29	35.03	36.44	35.05	34.42	0.420	0.665
Feed conversion ratio	1.89	1.84	1.86	1.82	1.72	0.039	0.724
Day 14 to 28							
Average daily gain (g/d)	54.86^b^	64.84^a^	65.95^a^	64.19^a^	68.26^a^	1.133	0.001
Average daily feed intake (g/d)	107.11	108.83	106.44	105.33	104.46	0.530	0.082
Feed conversion ratio	2.00^a^	1.70^b^	1.62^b^	1.65^b^	1.54^b^	0.036	<0.001
Day 1 to 28							
Average daily gain (g/d)	36.92^b^	42.03^a^	43.00^a^	41.80^a^	44.39^a^	0.540	<0.001
Average daily feed intake (g/d)	71.20	71.93	71.44	70.19	69.44	0.362	0.185
Feed conversion ratio	1.94^a^	1.72^b^	1.66^bc^	1.68^bc^	1.57^c^	0.026	<0.001

IgY, immunoglobulin Y; SA, alginate; CS, chitosan; SEM, standard error of the mean.

1) Each mean represents 10 replicate cages.

2) NC, basal diet; CH, basal diet+500 mg chlortetracycline/kg diet; NM, basal diet+50 mg non-microencapsulated IgY/kg diet; LM, basal diet + 600 mg microencapsulated IgY/kg diet; HM basal diet + 700 mg microencapsulated IgY/kg diet.

a–c Different superscript letters indicate significant differences in the rows (p<0.05).

**Table 3:** The effects of the IgY-loaded SA/CS/SA microcapsules on the relative organ weights and intestinal lengths in broilers^1)^

Item	Dietary treatment^2)^					SEM	p-value

	NC	CH	NM	LM	HM		
Relative weight (g/kg BW)							
Duodenum	8.91	8.58	8.95	8.62	8.55	0.114	0.699
Jejunum	15.92	15.65	15.76	15.11	15.13	0.206	0.640
Ileum	2.22	2.29	2.38	2.34	2.21	0.050	0.79
Spleen	1.22^c^	1.31^ab^	1.25^bc^	1.32^a^	1.36^a^	0.013	0.002
Thymus	2.81^b^	3.08^ab^	2.95^ab^	3.16^a^	3.21^a^	0.047	0.039
Bursa of Fabricius	2.00	2.07	2.03	2.13	2.15	0.025	0.225
Relative length (cm/kg BW)							
Duodenum	32.8	32.01	33.17	31.62	32.55	0.333	0.616
Jejunum	62.23	60.30	59.88	61.71	63.31	0.654	0.461
Ileum	11.09	11.15	10.59	10.64	10.84	0.165	0.768

IgY, immunoglobulin Y; SA, alginate; CS, chitosan; SEM, standard error of the mean; BW, body weight.

1) Each mean represents 10 individual broiler.

2) NC, basal diet; CH, basal diet+500 mg chlortetracycline/kg diet; NM, basal diet+50 mg non-microencapsulated IgY/kg diet; LM, basal diet + 600 mg microencapsulated IgY/kg diet; HM basal diet + 700 mg microencapsulated IgY/kg diet.

a–c Different superscript letters indicate significant differences in the rows (p<0.05).

**Table 4:** The effects of the IgY-loaded SA/CS/SA microcapsules on the morphology of broiler intestinal segments^1)^

Item	Dietary treatment^2)^					SEM	p-value

	NC	CH	NM	LM	HM		
Duodenum							
VH (μm)	1,365.0^b^	1,449.6^a^	1,377.9^b^	1,460.8^a^	1,491.8^a^	10.396	<0.001
CD (μm)	175.4	170.9	167.4	169.1	158.6	2.286	0.211
VH:CD	7.83^c^	8.53^bc^	8.28^bc^	8.76^ab^	9.45^a^	0.133	0.001
Jejunum							
VH (μm)	943.2^c^	971.3^bc^	951.5^c^	1,003.3^ab^	1,027.8^a^	7.910	0.001
CD (μm)	152.5	150.2	145.3	133.9	132.7	3.219	0.163
VH:CD	6.29^c^	6.63^bc^	6.74^bc^	7.58^ab^	7.92^a^	0.172	0.008
Ileum							
VH (μm)	974.2^b^	993.4^b^	1,004.4^b^	1,073.0^a^	1,110.4^a^	12.193	<0.001
CD (μm)	162.2	160.0	157.1	152.6	151.2	2.142	0.439
VH:CD	6.04^c^	6.29^c^	6.44^bc^	7.10^ab^	7.42^a^	0.134	0.002

IgY, immunoglobulin Y; SA, alginate; CS, chitosan; SEM, standard error of the mean; VH, villus height; CD, crypt depth.

1) Each mean represents 10 individual broiler.

2) NC, basal diet; CH, basal diet + 500 mg chlortetracycline/kg diet; NM, basal diet + 50 mg non-microencapsulated IgY/kg diet; LM, basal diet + 600 mg microencapsulated IgY/kg diet; HM basal diet + 700 mg microencapsulated IgY/kg diet.

a–c Different superscript letters indicate significant differences in the rows (p<0.05).

**Table 5:** The effects of the IgY-loaded SA/CS/SA microcapsules on the serum biochemical indices of broiler chickens^1)^

Item	Dietary treatment^2)^					SEM	p-value

	NC	CH	NM	LM	HM		
TP (g/L)	24.24	24.59	25.32	24.09	26.43	0.374	0.264
ALB (g/L)	12.84^c^	14.02^ab^	13.16^bc^	13.80^a^	14.40^a^	0.154	0.005
TC (mmol/L)	2.77	2.92	2.81	2.92	2.99	0.034	0.246
TG (mmol/L)	0.36	0.38	0.38	0.41	0.37	0.006	0.253
GLU (mmol/L)	10.37	10.58	10.87	10.87	10.69	0.109	0.568
ALP (U/L)	2,509.88	2,564.49	2,432.75	2,556.76	2,633.02	34.069	0.449

IgY, immunoglobulin Y; SA, alginate; CS, chitosan; SEM, standard error of the mean; TP, total protein; ALB, albumin; TC, total cholesterol; TG, triglyceride; GLU, gLucose; ALP, alkaline phosphatase.

1) Each mean represents 10 individual broiler.

2) NC, basal diet; CH, basal diet + 500 mg chlortetracycline/kg diet; NM, basal diet + 50 mg non-microencapsulated IgY/kg diet; LM, basal diet + 600 mg microencapsulated IgY/kg diet; HM basal diet + 700 mg microencapsulated IgY/kg diet.

a–c Different superscript letters indicate significant differences in the rows (p<0.05).

**Table 6:** The effects of the IgY-loaded SA/CS/SA microcapsules on serum immunoprotein levels in the broiler chickens^1)^

Item	Dietary treatment^2)^					SEM	p-value

	NC	CH	NM	LM	HM		
IgA (g/L)	2.34^c^	2.65^bc^	2.55^c^	2.92^ab^	3.09^a^	0.061	<0.001
IgG (g/L)	3.69^c^	4.43^b^	4.47^b^	4.62^ab^	4.92^a^	0.086	<0.001
IgM (g/L)	2.17^b^	2.46^ab^	2.47^a^	2.62^a^	2.77^a^	0.056	0.009

IgY, immunoglobulin Y; SA, alginate; CS, chitosan; SEM, standard error of the mean; IgA, immunoglobulinA, IgG, immunoglobulinG; IgM, immunoglobulin M.

1) Each mean represents 10 individual broiler.

2) NC, basal diet; CH, basal diet + 500 mg chlortetracycline/kg diet; NM, basal diet + 50 mg non-microencapsulated IgY/kg diet; LM, basal diet + 600 mg microencapsulated IgY/kg diet; HM basal diet + 700 mg microencapsulated IgY/kg diet.

a–c Different superscript letters indicate significant differences in the rows (p<0.05).

**Table 7:** The effects of the IgY-loaded SA/CS/SA microcapsules on serum antioxidants in the broiler chickens^1)^

Item	Dietary treatment^2)^					SEM	p-value

	NC	CH	NM	LM	HM		
MDA (nmol/mL)	3.23^a^	2.68^b^	2.74^b^	2.59b^c^	2.30^c^	0.070	<0.001
SOD (U/mL)	47.62^c^	58.56^b^	54.6^b^	56.16^b^	64.33^a^	1.043	<0.001
T-AOC (U/mL)	9.36^b^	9.49^b^	9.73^b^	9.95^b^	11.35^a^	0.167	<0.001
GSH-Px (U/mL)	702.08^c^	748.96^b^	755.59^b^	776.23^ab^	809.67^a^	8.515	0.001

IgY, immunoglobulin Y; SA, alginate; CS, chitosan; SEM, standard error of the mean; MDA, Malondialdehyde; SOD, Superoxide dismutase; T-AOC, total antioxidant capacity; GSH-Px, glutathione peroxidase.

1) Each mean represents 10 individual broiler.

2) NC, basal diet; CH, basal diet + 500 mg chlortetracycline/kg diet; NM, basal diet + 50 mg non-microencapsulated IgY/kg diet; LM, basal diet + 600 mg microencapsulated IgY/kg diet; HM basal diet + 700 mg microencapsulated IgY/kg diet.

a–c Different superscript letters indicate significant differences in the rows (p<0.05).

**Table 8:** The effects of the IgY-loaded SA/CS/SA microcapsules on the viable counts of *Escherichia coli*, Salmonella gallinarum, bifidobacteria and lactic acid bacteria in colon of broilers^1)^

Item (lg cfu/g)	Dietary treatment^2)^					SEM	p-value

	NC	CH	NM	LM	HM		
*Escherichia coli*	8.55^a^	7.64^b^	7.78^b^	7.52^b^	7.34^b^	0.104	0.001
Salmonella Gallinarum	8.41^a^	7.31^b^	7.46^b^	7.56^b^	7.69^b^	0.105	0.006
Lactic acid bacteria	8.05^c^	8.35^bc^	8.16^c^	8.56^b^	9.18^a^	0.085	<0.001
Bifidobacterium	9.50^b^	9.60^b^	9.57^b^	9.48^b^	10.01^a^	0.061	0.034

IgY, immunoglobulin Y; SA, alginate; CS, chitosan; SEM, standard error of the mean.

1) Each mean represents 10 individual broiler.

2) NC, basal diet; CH, basal diet + 500 mg chlortetracycline/kg diet; NM, basal diet + 50 mg non-microencapsulated IgY/kg diet; LM, basal diet + 600 mg microencapsulated IgY/kg diet; HM basal diet + 700 mg microencapsulated IgY/kg diet.

a–c Different superscript letters indicate significant differences in the rows (p<0.05).
